# Boron Chemicals in Drug Discovery and Development: Synthesis and Medicinal Perspective

**DOI:** 10.3390/molecules27092615

**Published:** 2022-04-19

**Authors:** Bhaskar C. Das, Nitesh K. Nandwana, Sasmita Das, Varsha Nandwana, Mohammed Adil Shareef, Yogarupa Das, Mariko Saito, Louis M. Weiss, Frankis Almaguel, Narayan S. Hosmane, Todd Evans

**Affiliations:** 1 Arnold and Marie Schwartz College of Pharmacy and Health Sciences, Long Island University, Brooklyn, NY 11201, USA; nitesh.nandwana@liu.edu (N.K.N.); sasmita.das@liu.edu (S.D.); varshanan3@gmail.com (V.N.); adilshareef.mohammed@liu.edu (M.A.S.); 2 Department of Medicine, Icahn School of Medicine at Mount Sinai, New York, NY 10029, USA; 3 Department of Surgery, Weill Cornell Medical College, Cornell University, New York, NY 10065, USA; tre2003@med.cornell.edu; 4 Nathan S. Kline Institute for Psychiatric Research, Orangeburg, NY 10962, USA; rojadas08@gmail.com (Y.D.); mariko.saito@nki.rfmh.org (M.S.); 5 Department of Pathology, Division of Parasitology and Tropical Medicine, Albert Einstein College of Medicine, Bronx, NY 10461, USA; louis.weiss@einsteinmed.org; 6 School of Medicine, Loma Linda University Health, Loma Linda, CA 92350, USA; falmaguel@llu.edu; 7 Department of Chemistry and Biochemistry, Northern Illinois University, DeKalb, IL 60115, USA; hosmane@niu.edu

**Keywords:** boron-containing drugs, boron-containing retinoids, aminoboronic acids, bezoxaboroles, lipogenic inhibitors, ALS, brain cancer, proteasome inhibitors

## Abstract

A standard goal of medicinal chemists has been to discover efficient and potent drug candidates with specific enzyme-inhibitor abilities. In this regard, boron-based bioactive compounds have provided amphiphilic properties to facilitate interaction with protein targets. Indeed, the spectrum of boron-based entities as drug candidates against many diseases has grown tremendously since the first clinically tested boron-based drug, Velcade. In this review, we collectively represent the current boron-containing drug candidates, boron-containing retinoids, benzoxaboroles, aminoboronic acid, carboranes, and BODIPY, for the treatment of different human diseases.In addition, we also describe the synthesis, key structure–activity relationship, and associated biological activities, such as antimicrobial, antituberculosis, antitumor, antiparasitic, antiprotozoal, anti-inflammatory, antifolate, antidepressant, antiallergic, anesthetic, and anti-Alzheimer’s agents, as well as proteasome and lipogenic inhibitors. This compilation could be very useful in the exploration of novel boron-derived compounds against different diseases, with promising efficacy and lesser side effects.

## 1. Introduction

**Figure 1:**
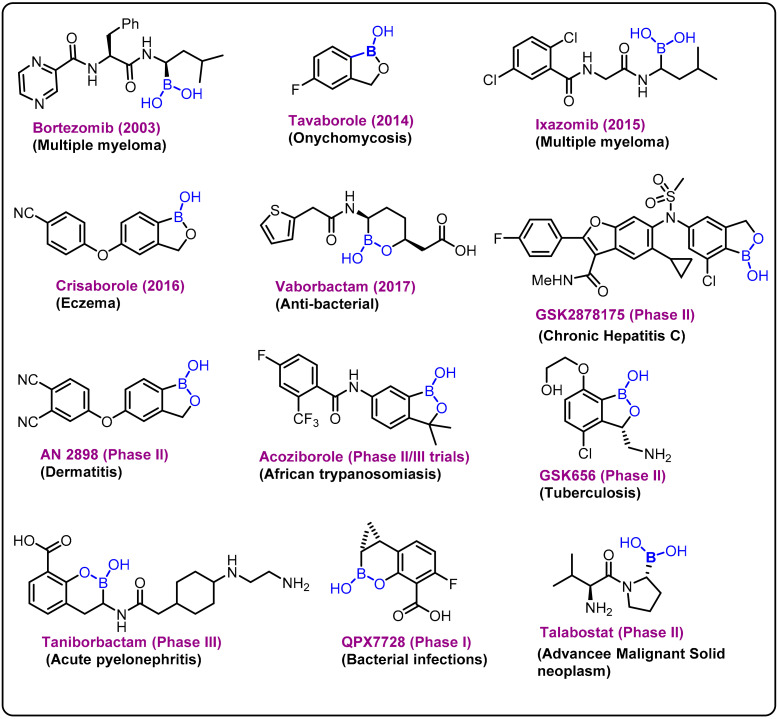
Examples and highlights of boron chemistry in drug discovery.

Due to its high electrophilic nature, the exogenous element boron and its compounds have been incorporated as key components in various drug candidates, and significant research has been reported over the last decade on the development of new boron-based scaffolds [1,2,3]. Boron has a remarkable ability to create stable electron-deficient three-center, two-electron bonds and strong covalent two-center, two-electron bonds. The covalent ligands present at the boron center can also form strong hydrogen bonds, which can improve effective coordination of active sites in a drug delivery process. In general, based on the literature, these bimodal interactions can be broadly classified into two categories: (i) primary and (ii) secondary interactions [4,5,6].The chemistry is evident from various studies on the behavior of boronic acid/ester present in various drug molecules [7,8,9,10,11,12]. Bortezomib (a dipeptide boronic acid), talabostat (a methanesulfonate salt of L-valinyl-L-boroproline), asborin (carbaborane analogue of aspirin), and tavaborole (a benzoxaborole) represent the remarkable advances in boron chemistry in the field of drug development (Figure 1). Bortezomib (Velcade)was the first boronic-acid-containing drug approved by the Food and Drug Administration (FDA) in 2003. This drug is a proteasome inhibitor that is used to treat multiple myeloma. Because of the overall beneficial properties of boronic acids, since the approval of Velcade, interest in boronic acids has grown in medicinal chemistry, leading to the discovery of two other drugs approved by drug authorities:ixazomib and vaborbactam. Ixazomib, approved by the FDA in 2015, is used to treat multiple myeloma and works via a mechanism similar to that of bortezomib. Vaborbactam, approved by FDA in 2017, is a β-lactamase inhibitor used in combination with antibiotics for the treatment of urinary, abdominal, and lung infections. Benzoxaborole-containing drugs tavaborole and crisaborole, approved by FDA in 2014 and 2017,are used for the treatment of onychomycosis and Eczema, respectively [1,13,14,15,16,17,18].

**Figure 2:**
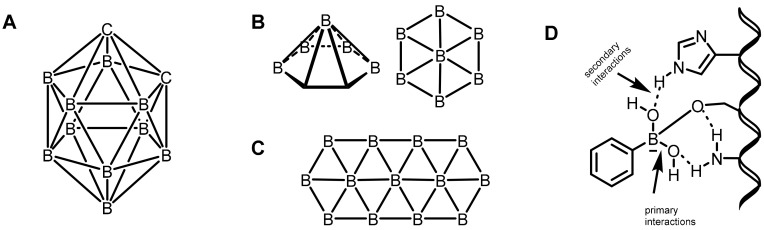
(**A**) *o*-carboranes. (**B**) Borophene layers. Top and side view of a B7 cluster as a constituting unit. (**C**) Fragment of a B36 borophene molecule. (**D**) Proposed primary and secondary interactions at the boron center.

Mechanistically, the primary interactions involve the boron–hydrogen–boron and Boron–Boron–Boronthree-center, two-electron bonds based on a large compilation of various boron clusters [19,20]. Based on this hypothesis, various carborane derivatives were found to be effective enzyme inhibitors. In particular, metallocarboranes have been found to be effective inhibitors of HIV protease enzymes (Figure 2A). However, the secondary coordinations are extended interactions from the ligands attached to the boron center, as shown in Figure 2B,C. Furthermore, the boron atoms can also be coordinated by strong electron-rich donors, as shown in Figure 2D. 

One relevant unique feature of the boron atom is its ability to capture neutrons. This has led to several drug discovery platforms. Mechanistically, the nuclear interaction between the boron-10 isotope and a thermal neutron generates an alpha particle, which recoils a lithium-7 atom with a 2.4 MeV. Before traveling one cell diameter, the alpha particle and lithium-ion expend their kinetic energy, resulting in precise cell death. If a compound containing the boron-10 isotope can selectively accumulate in tumor tissue, neutron irradiation can destroy tumor cells without causing substantial damage to normal tissues [21].

In this review, we will collectively provide a brief summary of successful boron drugs in medicinal chemistry. Boron drugs are versatile in addressing a multitude of human diseases (Table 1). 

## 2. Boron-Based Therapeutics

### 2.1. Boronic Acids/Esters/Aminoboronic Acids as Drug Motifs 

**Scheme 1:**
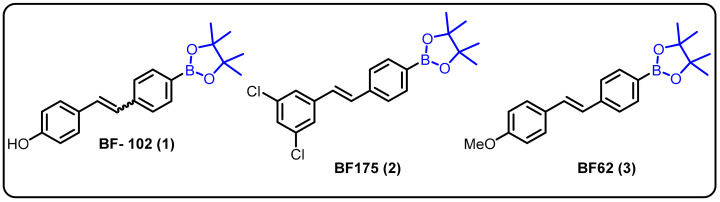
Boron-containing stilbene derivatives as potential drug candidates.

One of the most challenging target areas in the field of medicinal chemistry is heart disease. A high level of lipids in the blood (hyperlipidemia) considerably raises the risk of heart disease. This issue has been addressed at the level of transcriptional control for the regulation of lipogenic and cholesterogenic enzymes to maintain effective lipid metabolism. However, progress in treating hyperlipidemia has remained difficult to achieve over the years for two major reasons: (1) the molecular mechanism of lipid metabolism is still unknown and (2) some patients with hyperlipidemia do not respond to statin-related side effects. Therefore, it is necessary to develop novel approaches for the treatment of hyperlipidemia. In this regard, we explored boron-containing stilbene-based compounds and tested them for their potential as lipogenic inhibitors in mammalian hepatocytes. It was found that compound **1** showed a significant decrease in rate-limiting enzymes in the synthesis pathways for both fatty acids and cholesterol at the mRNA level [22]. Interestingly, it was also observed that the particular drug (BF102) has no significant toxicity in mice at the highest possible dose administered in our preliminary in vivo experiments (Scheme 1).

Dysregulation of lipid homeostasis has become a risk factor, leading to several disorders, such as obesity, hyperlipidemia, insulin resistance, fatty liver, and hypertension [69,70,71]. Among the known lipogenic regulators, sterol regulatory element-binding protein (SREBP) transcription factors are master regulators of lipid homeostasis. The Yang group described boron-based compoundBF175 (**2**) as a potential lead molecule for the treatment of human diseases caused by dysregulation of lipid homeostasis. BF175 (**2**) effectively inhibited SREBP target gene expression and lipid biosynthesis in cultured cells by blocking SREBP-TAD binding to MED15-KIX. However, other analogues, such as BF62 (**3**), failed to show significant activity [23]. In addition, it was the first small-molecule blocker of the physical interaction between MED15-KIX and SREBP-TAD.

**Scheme 2:**
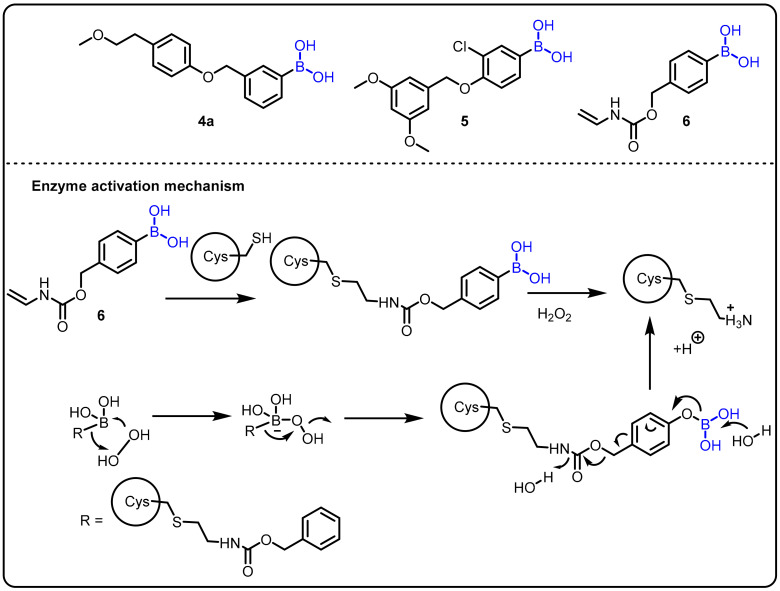
Boron drug for treatment amyotrophic lateral sclerosis.

A fascinating feature of boronic acids is their ability to act as a serine trap. Hixon et al. studied the influence of various boronic acids against autotoxin [72]. Structurally similar to serine hydrolases, autotoxin is a phospholipase implicated in cardiovascular disease. Autotoxin has a catalytic threonine residue but stabilizes the transient oxyanion through bimetallic coordination and is therefore inhibited by boronic acids. Following the fragment-based screening (FBS) protocol, in comparison to the compounds previously reported by the Kawaguchi group [73], new compounds **4**, **5**, and **6** were proven to be potent (Scheme 2).

Amyotrophic lateral sclerosis (ALS) is an incurable progressive nervous system disease that affects the spinal cord and nerve cells in the brain. It is linked to hyperactive superoxide dismutase (SOD1) and hypoactive angiogenin (ANG), which catalyzes the development of H_2_O_2_. Therefore, the chemical reactivity of H_2_O_2_, such as the oxidative cleavage of the boron–carbon bond in phenylboronic acid to produce boric acid (B(OH)_3_) and phenol, could be used in a physiological context. This phenomenon has been used as the basis of chemoselective probes for H_2_O_2_ in several cancer prodrug methods. Using this strategy, Hoang et al. introduced a novel synthetic boronic acid mask that restrains the ribonucleolytic activity of ANG [24].Compound **5** accumulates H_2_O_2_ and serves to selectively unmask the semisynthetic ANG for the treatment of amyotrophic lateral sclerosis (ALS) (Scheme 2).

**Scheme 3:**
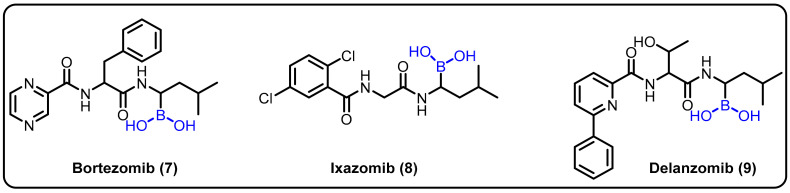
Boronic-acid-containing proteasome inhibitors.

Insights into peptide hydrolysis and methods for the development of next-generation proteasome-based cancer therapies can be elucidatedby understanding inhibitory mechanisms. Proteasome inhibition is proposed as an efficient strategy for restricting cancer growth, as well as in the treatment of tumors and hematological malignancies. In this regard, boronic acids have shown an interesting paradox for inhibiting various enzymes at different affected cell sites. Boronic acid inhibitors are particularly noteworthy because bortezomib (**7**) was the first proteasome inhibitor to be licensed for the treatment of multiple myeloma in 2004 [25]. Insights into the catalytic mechanisms of inhibition necessitate a revised description of the active proteasome site. Ixazomib (**8**) was approved as an orally bioavailable inhibitor to treat the same disease in 2015, [26] and delanzomib (**9**) is presently in clinical trials for this purpose and to treat autoimmune disease [27]. In 2016, Chari et al. revealed the crystal structures of the native human 20S proteasome and its complexes with inhibitors [74] (Scheme 3).

**Scheme 4:**
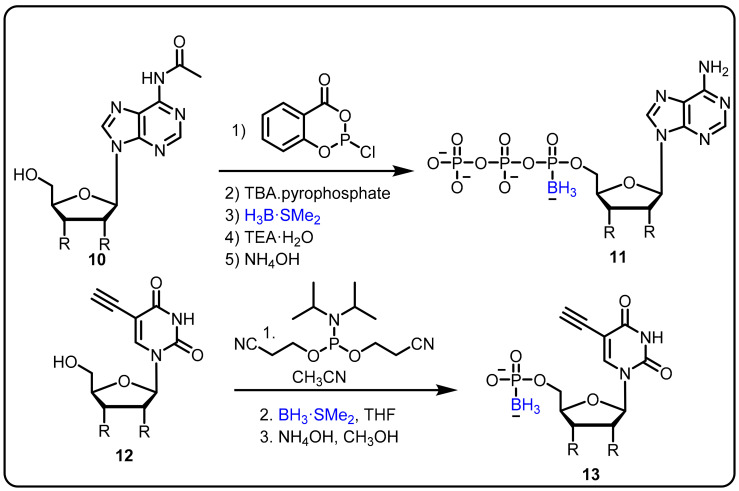
Boranophosphate nucleosides as an inhibitors of HIV reverse transcriptase (RT).

Various nucleoside analogues, such as 3-thia-2,3-dideoxycytidine (3TC), 3-azido-3′-deoxythymidine (AZT), and 2,3-didehydro-2,3- dideoxythymidine (d4T) have been found to actas antiviral agents targeted at the reverse transcriptase (RT) of HIV [75]. The absence of a 3′ hydroxyl group supportsviral DNA chain termination. Three major resistance mechanisms operate at the RT active sites for viral RT gene mutation to control DNA chain termination under therapeutic conditions; these can be described as: repair of the analogue-terminated DNA, discrimination of the analogue by a decreased affinity (Kd) for RT, and a decreased polymerization rate (kpol) [76]. In this regard, *α*-boranophosphate nucleosides (*α*-BH_3_-dNTPs), as analogues with one non-bridging oxygen on the phosphate substituted by a BH_3_^¯^ group, evolved as one efficient strategy to circumvent this resistance [77]. Canard et al. reported BH_3_-nucleotide analogues as HIV-1 RT inhibitors, having shown the ability to overcome reverse transcriptase-mediated drug resistance [78]. Furthermore, Shaw et al. explained the design and synthesis of cordycepin and 2′-*o*-methyl α-*P*-borano triphosphate by using a one-pot phosphorochloridite reaction under mild reaction conditions (Scheme 4) [28,79]. The α-*P*-borano compounds in which α-phosphateoxygen was replaced by borane were tested in cell culture and displayed potential as effective viral polymerase inhibitors. The overall goal of this project was to improve the antiviral potency of chain-terminating sugar and base-modified purine nucleoside versus the hepatitis C viral RNA-dependent RNA polymerase by using a combination of α-*P*-borano and nanogel drug delivery technology. Subsequently, a potent thymidylate synthase inhibitor was obtained using a similar boronophosphate scaffold. 

**Scheme 5:**
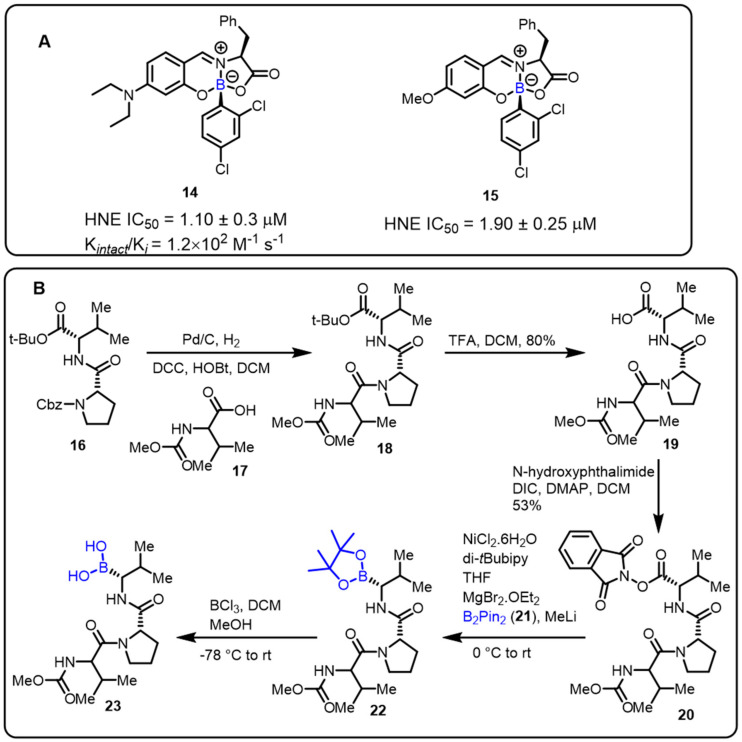
(**A**) Borate complexes. (**B**) Baran’s protocol for the synthesis of HNE inhibitors.

The Gois group described a novel one-pot approach for the synthesis of boron-containing bioactive scaffolds by the reaction of aryl boronic acids, salicylaldehyde, and amino acids to identify effective enzyme inhibitors. The developed protocol was applied for the discovery of human neutrophil elastase (HNE) inhibitors. Compounds **14** and **15** were found to be the most efficient among the tested substances, with IC_50_ values of 1.1 and 1.9 μM, respectively, against HNE [29]. Moreover, adetailed mechanistic investigation revealed that imine alkylation and lactone ring-opening are key features ofthe mechanism of inhibition (Scheme 5).

Furthermore, Baran and colleaguesdeveloped an easy decarboxylative-coupling strategy to obtain a highly biologically important boron-containing molecule (Scheme 5) [80]. This method achievedtremendous output in synthesizing Velcade, and one suchhuman neutrophil elastase (HNE) inhibitor compound is now in phase II clinical trials. Amino boronic acids, as potent enzyme inhibitors, can be effectively synthesized in a conventional process via the decarboxylation of the corresponding amino acids. 

**Scheme 6:**
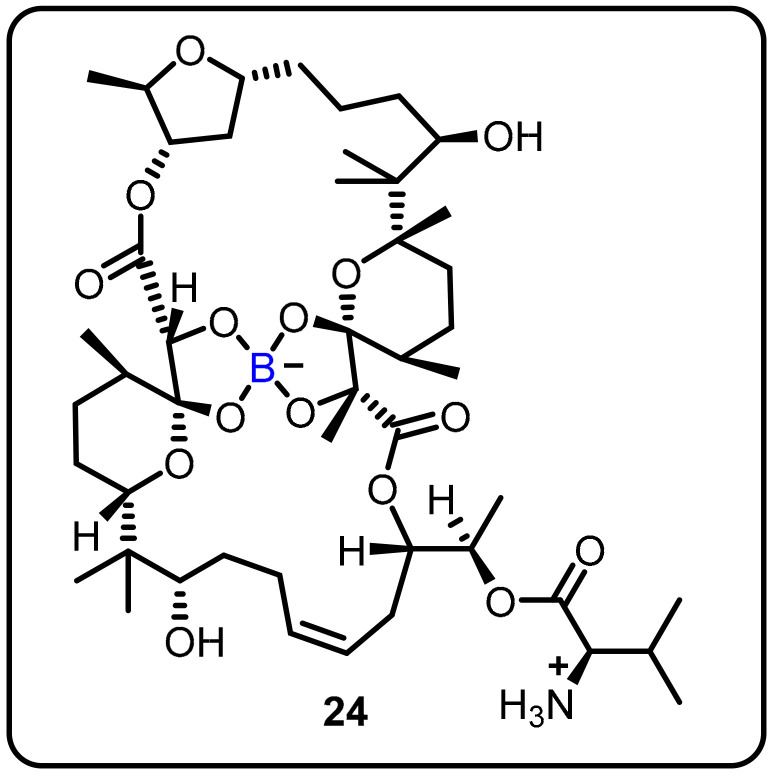
Boromycin.

The anti-TB activity of boromycin (**24**) against mycobacterial persisters without causing resistance was described by Dick and colleagues. Boromycin was discovered to be active against Gram-positive bacteria and served as a potassium ionophore after being isolated from *Streptomy cesantibioticus.* Boromycin inhibited mycobacterial growth, with high bactericidal efficacy against both growing and non-growing drug-tolerant persister bacilli (MIC_50_ = 80 Nm) [30]. Upon exposure to boromycin, intracellular ATP levelsand the membrane potential were reduced, and cytoplasmic proteins were leaked. The addition of KCl to the medium, which was consistent with boromycin working as a potassium ionophore, inhibited its antimycobacterial activity. Boromycin was also shown to have good cytotoxicity and hemolytic activity (CC_50_ = 30 μM, HC_50_ = 40 Mm), as well as a selectivity index of more than 300 (Scheme 6).

**Scheme 7:**
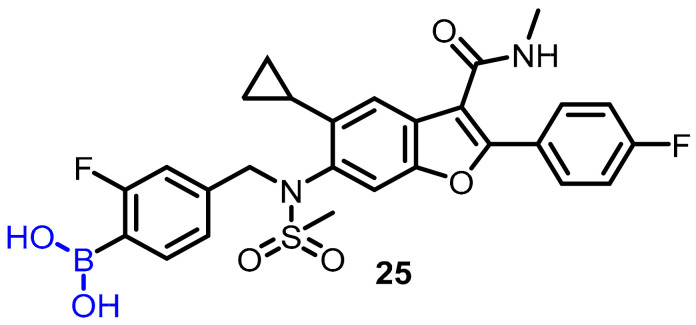
Potent boronic-acid-derived inhibitor of HCV.

Hepatitis C virus (HCV) produces chronic HCV infection in millions of people around the globe, resulting in liver cirrhosis and hepatocellular carcinoma. In this regard, Maynard et al. proposed using boronic acid as a pharmacophore to targeta subset of non-structural protein 5B (NS5B) polymorphs and resistant mutants [33]. The authors designed and synthesized a series of compounds, and among them, compound **25** was found to be an effective potent inhibitor of NS5B polymerase; the authors described boronic-acid-containing moiety as a critical pharmacophore. The potency against key resistant mutants was optimized to afford clinical candidate **25.** The most potent compound (**25**) showed slow binding kinetics with isolated GT1b 316N protein, which was characterized bya division half-life of >40 h (Scheme 7), and also blocked the initial step of the polymerase RNA replication cycle. In hepatitis C virus-infected patients, compound **25** induced a significant reduction in serum HCV ribonucleic acid (RNA) after a single dose of 420 mg (−1.33 log_10_ copies/mL) compared to the placebo (−0.09 log_10_ copies/mL) 24 h post-dose (Scheme 7).

#### Breakthrough 1

Bortezomib (**7**) was the first proteasome inhibitor breakthrough approved by the FDA in 2003 for the treatment of newly diagnosed multiple myeloma patients and mantle cell lymphoma [34,35,36,37]. Bortezomib has displayed several clinical benefits, either alone or in combination with other drugs, inducing radio/chemosensitization and overcomingdrug resistance. The overexpression of NOXA is one of the important mechanisms associated with bortezomib’s anticancer activity (NOXA is a proapoptotic protein that interacts with the antiapoptotic Bcl-2 subfamily proteins Bcl-XL and Bcl-2 to cause apoptotic cell death in malignant cells). Another mechanism of bortezomib is suppression of the nuclear factor-κb (NF-κB) signaling pathway, which leads to the downregulation of its antiapoptotic target genes. The majority of bortezomib’s success has been achieved in hematological malignancies, whereas its effect on solid tumors has been less promising. Further significant progress with bortezomib was described by Matsuoka et al. highlighting the growth inhibition of adult T-cell leukemia both in vivo and in vitro [38]. Bortezomib suppressed tumor progression in SCID mice bearing tumors, suggesting that bortezomib has been efficient against adult T-cell leukemia (ATL) cells in vivo. These findings indicate that bortezomib is extremely efficient against ATL cells in vitro and in vivo by induction of apoptosis, and its therapeutic potential could improve the prognosis of patients with this lethal disease.

**Scheme 8:**
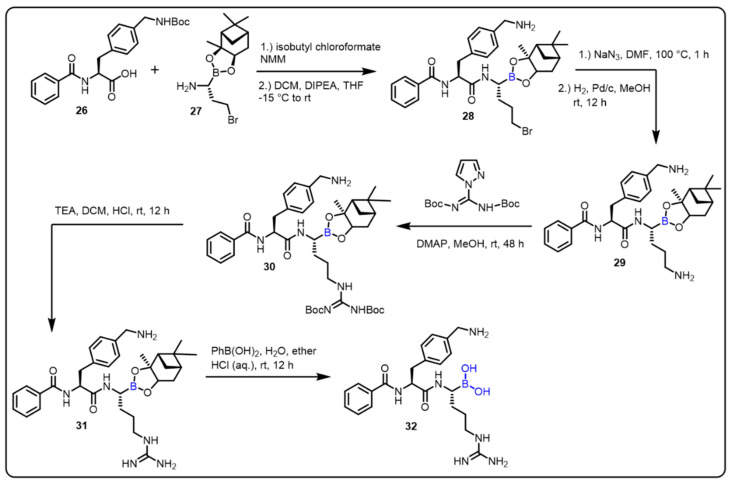
Peptide-boronic acid inhibitors of flaviviral proteases.

The Klein group described the boron-based moiety with effective candidates against Zika, West Nile, and Dengue virus proteases. Flaviviruses are responsible for these infections; therefore, inhibition of flavivirus proteases is an interesting approach for the treatment of all three diseases. The authors achieved a thousand-fold affinity by the presence of C-terminal boronic acid moiety into dipeptidic inhibitors, such as protease **32**. The desired compound (**32**) displayed K*_i_* values in the two-digit nanomolar range, and no cytotoxicity was observed, while inhibiting virus replication. In vitro studies revealed corresponding K*_i_* values in the range of K*_i_* = ~0.05, IC_50_ = 0.066 μM (for Dengue), K*_i_* ~0.08, IC_50_ = 0.11 μM (for West Nile), K*_i_* ~0.04, IC_50_ = 0.25 μM (for Zika). (Scheme 8) [40]. Furthermore, the authors explained that boronic acid **32** formed a hydrogen bond with the oxygen in the active site of the flaviviral protease’s Ser135 residue.

### 2.2. Benzoxaborole as Drug Motifs

In 1957, benzoxaboroles were designed and synthesized for the first time [81]. Long-term research has revealed that these oxaborole rings are extremely stable and also have high hydrolytic resistance of the boron–carbon bond when compared to the corresponding boronic acids. Moreover, benzoxaborolesare usually more soluble in water than boronic acids. As the boron compounds exhibit a diverse electronic orientation in different forms, benzoxaboroles are also sensitive to structural changes induced by substitution. The ranges of geometric parameters reveal that the exocyclic B-O bond varies in the range of 1.337 Å–1.372 Å, but the endocyclic bond changes between 1.372 Å and 1.412 Å. The lengths of the C-B and C-O bonds also vary widely, ranging from 1.496 Å to 1.565 Å and 1.436 Å to 1.488 Å, respectively. Importantly, these differences are the result of intermolecular interactions in the crystal lattice, as well as the electronic structure of the substituents [82,83,84].

**Scheme 9:**
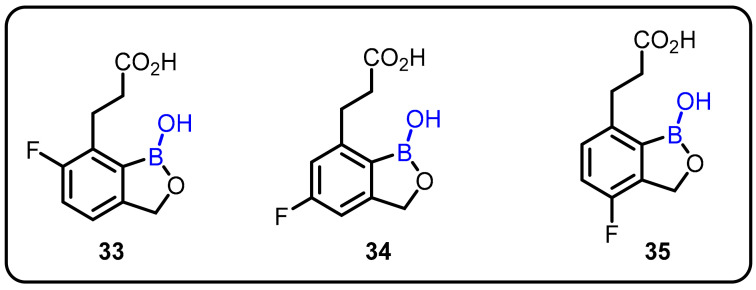
Fluoro-substituted 7-(2-carboxyethyl)-1,3-dihydro-1-hydroxy-2,1-benzoxaboroles as antimalarial agents.

Malaria: 

Malaria is a human tragedy estimated to have killed half of all people who ever lived; currently, the disease claims the lives of hundreds of thousands of young children every year. In 2013, Malaria represented a parasite infection that caused an estimated 198 million clinical cases and 584,000 deaths worldwide, with the majority of cases occurring in children under the age of five. *Plasmodium falciparum*, the most common risk parasite, is transmitted to humans by infected mosquitoes and is responsible for the vast majority of severe morbidity and mortality. Zhang et al. designed and synthesized a series of novel benzoxaborole derivatives by varying different substituents on the benzene ring. All the synthesized compounds were evaluated for their antimalarial activity, and three compounds—**33**, **34**, and **35**—were found to be effective against *Plasmodium falciparum*, with IC_50_ values of 0.209, 0.061, and 0.209 μM, respectively (Scheme 9). Notably, investigation into the structure–activity relationship (SAR) revealed that boron is essential for antimalarial activity [41].

**Scheme 10:**
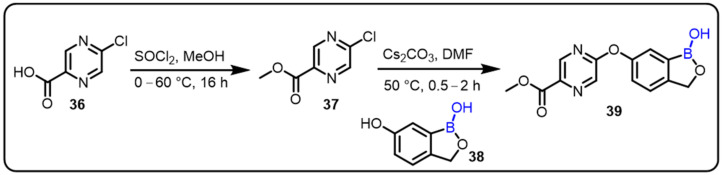
6-(2-(Alkoxycarbonyl)pyrazinyl-5-oxy)-1,3-dihydro-1-hydroxy-2,1-benzoxaboroles as antimalarial agents.

Recently, additional boron-containing benzoxaboroles have displayed potent activity against *P*. *falciparum*; the best activity among tested compounds was obtained with an IC_50_ at 26 nM [41,42,43,44]. Yang and colleagues synthesized a series of 6-hetaryloxy benzoxaborole compounds and evaluated their antimalarial activity against W2 and 3D7 strains of *P. falciparum*. SAR was investigated by varying different substituents on the pyrazine ring to assess the changes in antimalarial activity. SAR studies revealed that6-(2-(alkoxycarbonyl)pyrazinyl-5-oxy)-1,3-dihydro-1-hydroxy-2,1-benzoxaboroles was highly potent, with IC_50_ = 0.2−22 nM against cultured *Plasmodium falciparum* W2 and 3D7 strains. Moreover, compound **39** also displayed excellent in vivo efficacy against *P. berghei* in infected mice (ED90 = 7.0 mg/kg) (Scheme 10) [45].

**Scheme 11:**
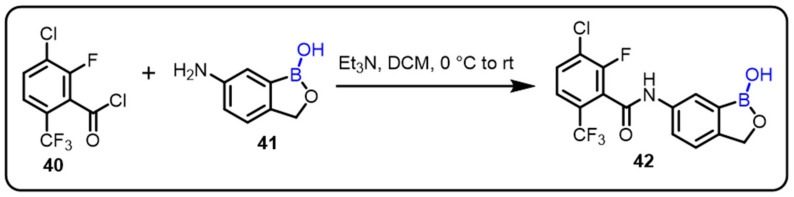
AN4161 as an anti-inflammatory agent.

Rheumatoid arthritis (RA), inflammatory bowel disease (IBD), and psoriasis: 

Therapeutics targeting tumor necrosis factor-α (TNF-α) and interleukin (IL) 1 and 6 are used to treat rheumatoid arthritis (RA), inflammatory bowel disease (IBD), and psoriasis. Due to its demand and compatibility, various structure–activity relationships of 6-(benzoylamino)benzoxaborole analogues were examined for the inhibition of TNF-α, IL-1β, and IL-6 from lipopolysaccharide-stimulated peripheral blood mononuclear cells. Interestingly, compound **42** showed potent activities against all three cytokines, with IC_50_ values between 0.19 and 0.50 μM [48]. The drug candidate also inhibited LPS-induced IL-6and TNF- α elevation and enhanced collagen-induced arthritis in mice. Compound **42** (AN4161) was observed to be a promising lead as a novel anti-inflammatory agent with a favorable pharmacokinetic profile (Scheme 11).

**Scheme 12:**
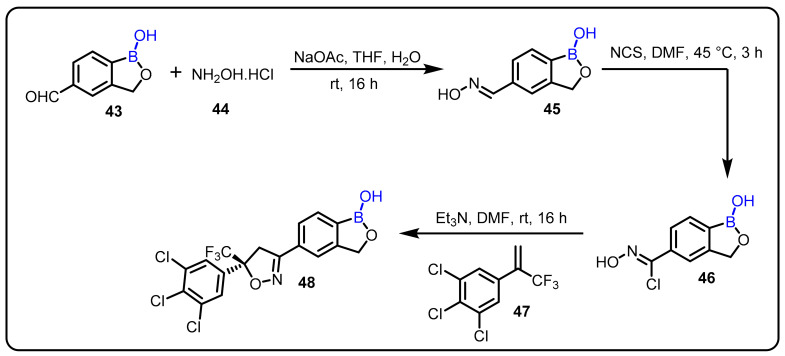
Ectoparasiticide activity of an isoxazoline-containing boraxozole.

Ticks and fleas: 

Zhang et al. designed and synthesized a series of isoxazoline benzoxaborole derivatives in an investigation to assess ectoparasiticide activity against ticks and fleas [46]. Compound **48** demonstrated 97.6% therapeutic efficacy after 24 h, with 95.3% residual efficacy against American dog ticks (*Dermacentor variabilis*) on day 30 and 100% residual efficacy against cat fleas (*Ctenocephalides felis*) on day 32 after a single oral dose of 50 mg/kg in dogs. (Scheme 12).

**Scheme 13:**
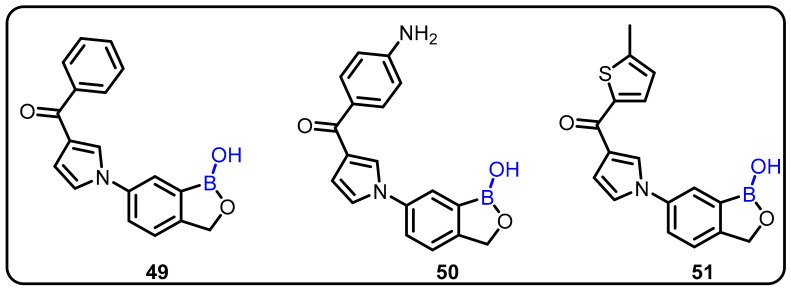
Antitrypanosomal activity of 6-pyrrolobenzoxaborole scaffold.

Anti-trypanosomal activity: 

The development of a new series of 6-pyrrolobenzoxaboroles derived from 6-indolylbenzoxaboroles significantly improved overall antitrypanosomal activity [85,86,87]. SAR of this new class of oxaboroles revealed that interchanging the substituents from the pyrrolyl C(2′) to the C(3′) provided significantly more efficient antitrypanosomal 6-pyrrolobenzoxaboroles, with IC_50_ values as low as 0.03 μg/mL and good cytotoxicity profiles. The authors proposed that this could be effective against African trypanosomiasis, in which a fatal parasitic infection is caused by the protozoan *Trypanosoma brucei*. The synthesized compounds were evaluated for their antiparasitic activity and have shown good antiparasitic IC_50_ values, ranging from 4.02 to 0.03 μg/mL, with satisfactory cytotoxicity profiles. Among the tested compounds, compounds **49**, **50**, and **51** wererevealed to cure parasitic infection in a murine acute infection model, with complete clearance of the parasites in the blood (Scheme 13) [47].

**Scheme 14:**
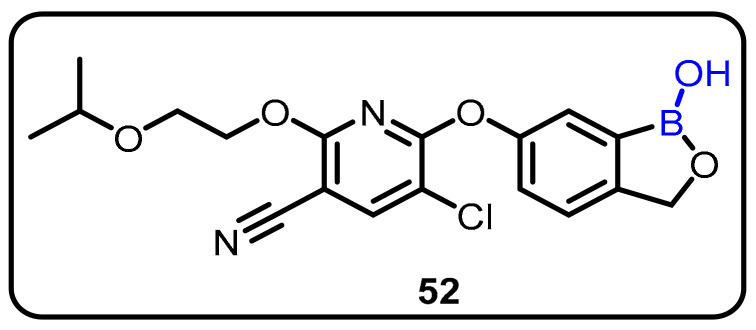
AN6414, a novel boron-containing PDE4 inhibitor.

Inflammatory diseases:

During drug discovery, identification of in vivo metabolites is of great interest in order toprovide a more complete picture of the metabolic profile to enable early safety assessment of metabolites. The Liu group described early rapid identification of in vivo rat metabolites of AN6414. AN6414 (**52**) was proposed a novel benzoxaborole for the treatment of inflammatory diseases. Compound **52** exhibited potent in vitro inhibitory activity, with IC_50_ values of 5.2 nm and 2.5 nm against PDE4 enzyme and TNFα- secretion, respectively, in human PBMCs [49]. Using rat plasma samples from a toxicokinetic investigation at an oral dose of 30 mg/kg, the authors demonstrated efficient and selective core-structure-relevant precursor scans and daughter ion scans and identified 10 significant phase I and phase II metabolites. In addition, in vivo study of LPS-induced plasma TNFα levels revealed an ED_50_ of 0.15 mg/kg, which is a much better dosage factor than that of roflumilast (7.5 mg/kg). The major metabolites of AN6414 underwent oxidative deboronation, protodeboronation, oxidation products, and their sulfate-conjugated species in a sequential manner. Moreover, the authors also described the toxicity profile using the software program DEREK, which revealed that the identified potential metabolites were safe (Scheme 14).

**Scheme 15:**
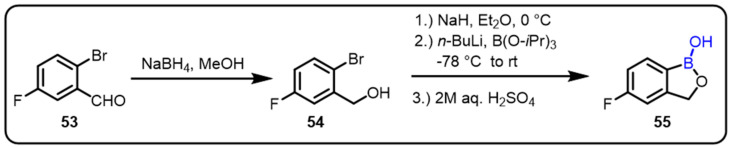
Synthesis oftavaborole: antifungal drug.

Anti-fungalDrugs: 

Tavaborole (**55**), a low-molecular-weight oxaborole antifungal drug developed by Anacor Pharmaceuticals Inc., was approved by theFDA in 2014 for the treatment of onychomycosis of the toenail. Tavaborole functions as an antifungal agentby inhibiting cellular protein via blocking of yeast cytoplasmic leucyl-aminoacyl transfer RNA (tRNA) synthetase. The boron atom of the oxaborole ring binds to the 20, 30-hydroxy groups on the tRNA 30-terminal adenosine, leading to the formation of a stable tRNALeu-tavaborole adduct, which inhibits leucyl-tRNALeu synthesis and protein synthesis. In addition, tavaborole analogues in which the boron atom in the oxaborole ring was replaced with carbon had no activity, suggesting that the boron atom in the oxaborole ring is required for the drug’s fungal inhibition mechanism (Scheme 15) [51].

**Scheme 16:**
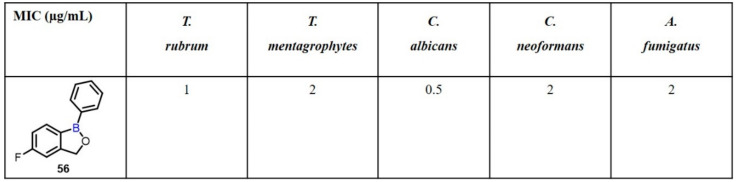
AN2690 for the potential treatment of onychomycosis.

Onychomycosis:

Onychomycosis is a progressive fungal infection of the nail unit that causes toenail and fingernail destruction and abnormality [88]. Dermatophytes, a type of fungus that lives on the skin, hair, and nails and generates other cutaneous fungal infections, such as athlete’s foot, are the primary cause of onychomycosis. The most predominant dermatophytes are *Trichophyton rubrum* and *Trichophyton mentagrophytes*, which represent 60–90% of all cases. In this regard, Baker et al. synthesized a series of benoxaborole derivatives bearing aryl, heteroaryl, or vinyl substituents from 2-bromo-5-fluorobenzaldehyde or 2-bromobenzyl alcohol. The synthesized compounds were evaluated for their antifungal activity.Among them, compound **56** displayed a potent inhibitory activity against *T. rubrum*, *T. mentagrophytes*, *C. albicans*, *C. neoformans*, and *A. fumigatus*, with MIC values in the range of 0.25–1 µg/mL (Scheme 16) [89].

**Scheme 17:**
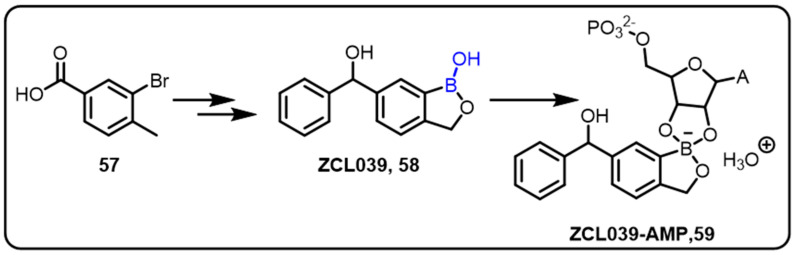
A potent benzoxaborole-based anti-pneumococcal agent targeting leucyl-tRNA synthetase.

Pneumonia:

Pneumonia is the leading cause of mortality and morbidity among children worldwide, according to the World Health Organization, killing more than two million children each year [90].The Wang group successfully demonstrated benzoxaborole-based compounds as antipneumococcal agents by targeting leucyl- tRNA synthetase [52]. The compound ZCL039 (**58**), a benzoxaborole-based derivative of tavaborole (AN2690), displayed potent inhibitory activity against *S. pneumonia* and *E. coli*, with MIC values of 5 and 60 mg/mL, respectively. The authors also described a benzoxaborole-based antipneumococcal drug with an OBORT (oxaborole tRNA trapping) mechanism, which was emphasized by the crystal structure of ZCL039 bound to SpLeuRS-CP1. When compared to human cytosolic LeuRS, ZCL039 (**58**) inhibited SpLeuRS aminoacylation, with a 150-fold higher sensitivity (Scheme 17).

**Scheme 18:**
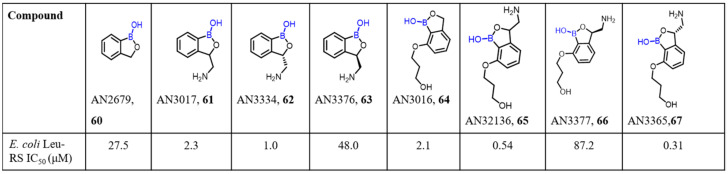
Benzoxaborole derivatives as antibacterial agents.

Multidrug bacterial resistance: 

In recent years, the rise in the prevalence of Gram-negative antimicrobial resistance has become a concerning factor. Gram-negative bacteria cause approximately 70% of infections in intensive care units [91]. A growing number of bacterial isolates are responsible for resistance to currently available antibiotics. Therefore, considerable effort has been directed to thedevelopment of new classes of antibiotics by the modification of existing drugs to overcome the resistance mechanism of bacteria. As described above, inhibition of aminoacyl-tRNA synthetase and leucyl-tRNA synthetase (LeuRS) in fungi has been demonstrated through an oxaborole tRNA-trapping (OBORT) mechanism [51]. In this regard, the Plattner group described a new series of boron-based antibiotics [53] that were tested for their antibacterial activity. AN3365 (**67**) was foundto beactive against *E. coli* LeuRS, with an IC_50_ value of 0.31 μM. The lead compound (**67**) was also found to be activeagainst Gram-negative bacteria, including *Enterobacteriaceae*-bearing NDM-1, KPC *carbapenemases*, and *P. aeruginosa.* Moreover, compound **67** has a good murine pharmacokinetics profile and was found to be efficacious against *E. coli* and *P. aeruginosa* in murine thigh infection models (Scheme 18).

**Scheme 19:**
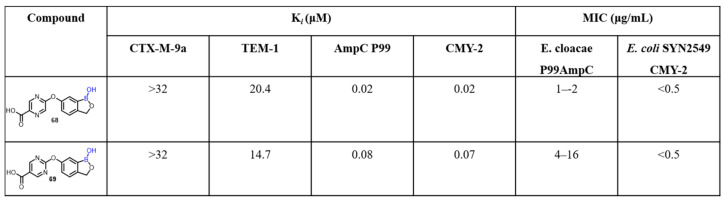
Benzoxaboroles as a new class of β-lactamase inhibitors.

Previous findings suggest that structural variation plays a crucial role in SAR studies, as well as in the overall activity of the drug candidate. As we have discussed in this review, the heteroaryls tethered at the sixth position of benzoxaborole moiety are effective. In this regard, Xia et al. synthesized a library of novel benzoxaborole β-lactamase inhibitors. The synthesized compounds were screened against enzyme TEM-1 and AmpC from *Enterobacter cloacae* P99 and showed promising broad-spectrum inhibitory activity. The 6-aryloxy benzoxaboroles (**68** and **69**) showed potent inhibition of AmpC P99 and CMY-2, with K*_i_* values in the lower nanomolar range (Scheme 19) [55]. SAR studies demonstrated variation of different ether linkers using different substitution patterns in the C6 position of benzoxaboroles. The authors also described the synthesis of heteroaryl analogues to investigate the effect of lipophilicity on antibacterial activity. Compound **68** restored antibacterial activity of ceftazidime against *Enterobacter cloacae* P99-expressing AmpC, a class C β-lactamase enzyme.

**Scheme 20:**
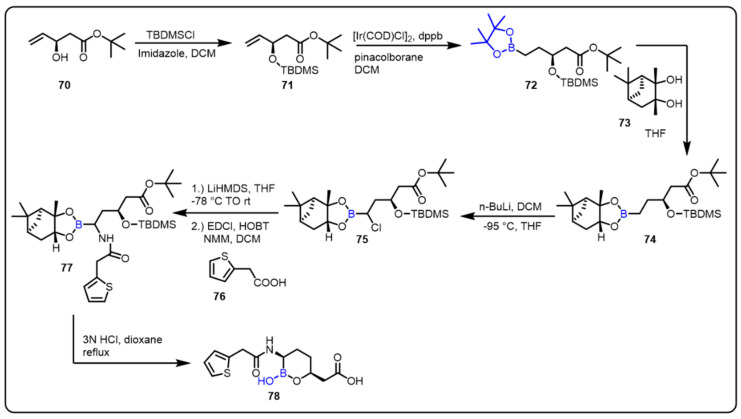
Cyclic boronic acid as a β-lactamase inhibitor (RPX7009).

The Dudley group described a new series of serine β-lactamase inhibitors bearing a cyclic boronic acid pharmacophore to find a potent inhibitor of serine carbapenemase enzymes. All the synthesized compounds were tested in biochemical and whole-cell assays. Among all tested compounds, compound **78** was found to be the most potent inhibitor of serine carbapenemases, mainly the *Klebsiella pneumoniae* carbapenemase (KPC), without inhibition of mammalian serine proteases. Moreover, compound **78** showed similar pharmacokinetics in the rat model to β-lactam antibiotics, including carbapenems. Notably, in vitro and in vivo studies revealed that RPX7009 (**78**) has broad-spectrum inhibitory activity [92]. Combined with a carbapenem, compound **78** was a promising drug candidate for the treatment of multidrug-resistant, Gram-negative bacteria (Scheme 20). 

### 2.3. Carboranes as Drug Motifs

Carboranes are boron, carbon, and hydrogen-based pseudoaromatic polyhedral clusters. Theywere introduced into biological systems as pharmacores in 1961 by Soloway et al. [93,94,95,96]. Carboranes are used as a rich source of boron for Boron Neutron Capture Therapy (BNCT) [97]. Carboranes have been valuable bioisosteres for phenyl groups and adamantane because their size and lipophilicity are comparable, but their remarkable structures allow for novel drug development frontiers to be explored. Another important feature of carboranes inmedicinal chemistry is their ability to convert the neutral, lipophilic closocluster into the anionic, hydrophilic nido species in a single synthetic step. Carborane functionalizations via various synthetic methodologieshave been of significant interest in recent years, which can be attributed to their remarkable stability towards moisture and biological degradation, as well astheir low toxicity. More notably, they facilitate transport across the blood–brain barrier (BBB), which is a significant challenge for the development of drugs for the central nervous system (CNS).

**Scheme 21:**
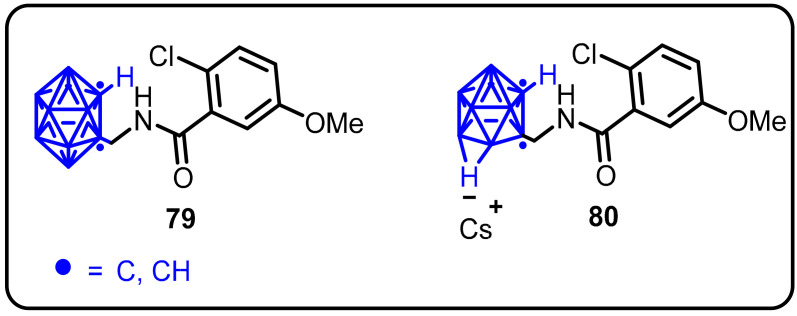
Novel P2X7 receptor antagonist with antidepressant activity.

The first CNS-active carborane was identifiedby Wilkinson et al. as a novel P2X7 receptor antagonist with antidepressant activity. This discovery wasa major contribution to the emerging field of using carboranes as pharmacophores in drug discovery. The closo-1,2-carborane (**79**) revealed unique advantages over adamantane due to its strong exogenous substrate, which delivers excellent metabolic stability and easily converts to its respective anionic nido-carborane cluster **80 [56]**. The notable in vivo antidepressant activity of Cs·46 was compatible with CNS P2X7R inhibition.This wasthe first report of a carborane molecule with CNS-modifying activity, opening the way for carboranes to be used as templates for the development ofCNS drugs in the future (Scheme 21).

**Scheme 22:**
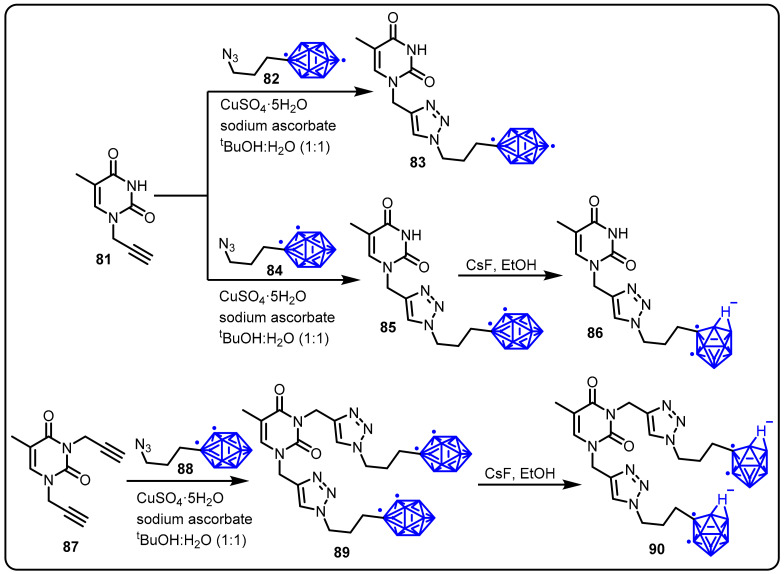
Synthesis of thymine-linked lipophilic 1,12-dicarba-closo-dodecaborane and 1,2-dicarba-closo-dodecaborane derivatives.

1,2,3-triazoles have a broad range of applications in the field of synthetic, medicinal, and material chemistry. Sharpless [98] and Meldal [99] presented a milestone for the region-selective synthesis of 1,4-disubstituted triazole by the reaction of azides with alkynes in the presence of copper salt. This kind of reaction is known as copper-catalyzed azide–alkyne cycloaddition (CuAAC) or click reaction. Olejniczak and colleagues developed a series of novel thymine derivatives, such as lipophilic, electron-neutral 1,2-dicarba-closo-dodecaborane; 1,12-dicarba-closo-dodecaborane;and hydrophilic 7,8-dicarba-nido-undecaborate anions, by the reaction of N^1^-propargylthymine or N^1^,N^3^-bispropargylthymine with 1-(3-azidopropyl)-1,2-dicarbacloso-dodecaborane through a copper-catalyzed 1,3-dipolar cycloaddition reaction [31]. The synthesized compounds were evaluated in vitro against *Mycobacterium tuberculosis* thymidylate kinase (TMPKmt) and as inhibitors of mycobacteria growth in culture against pathogenic *Mycobacterium tuberculosis* (*M. tuberculosis*) and saprophytic *Mycobacterium smegmatis* (*M. smegmatis*) strains. Among all tested compounds, compound **90** was found to have the most potent TMPKmt inhibitor activity, exhibiting a K*_i_* value of 1.5 mM (Scheme 22). In addition, compounds **85** and **89** inhibited *M. smegmatis* proliferation at a concentration of 100 mg/mL, with MIC values of 0.25 mM and 0.15 mM, respectively.

**Scheme 23:**
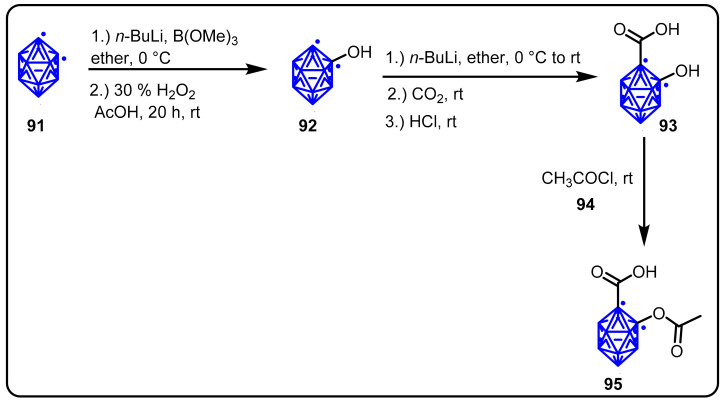
Asborin: the carbaborane analogue of aspirin.

Aspirin, the acetyl ester of salicylic acid, was discovered in the late 1800s and immediately came to fame as one of the most well-known medications in the world. Its fame arises from its capacity to treat a wide range of symptoms, such as fever, inflammation, and pain. Hawkins et al. altered aspirin by substituting the phenyl ring with a three-dimensional and highly hydrophobic carbaborane cluster. Asborin (**95**) showed cyclooxygenase (COX) inhibition and isoform selectivity in an enzyme-linked immunosorbent assay (ELISA)-based COX inhibitor screen [100]. Asborin was slightly less active than aspirin in the free enzyme system, but the activity in a whole-blood or cell-based assay was different because the enzyme entry in living systems was hidden in the interior of the membrane [85] COX-1 activity was estimated to be 50% inhibited at an asborin concentration of 500 mm, and COX-2 activity was decreased to a lesser extent, by 28%. As a result, asborin, like aspirin, could inhibit COX enzymes (Scheme 23) [101].

**Scheme 24:**
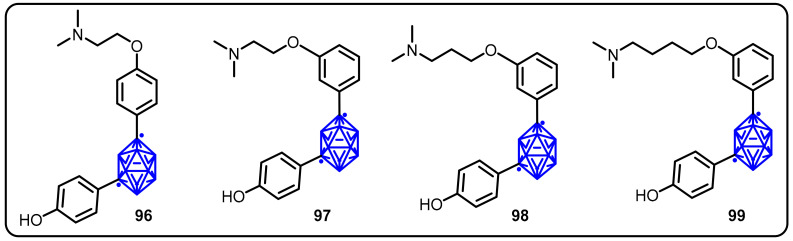
*m*-Carborane-containing estrogen receptor partial agonists as SERM candidates.

In 2015, Ohta et al. developed a novel m-carborane-containing selective estrogen receptor modulator (SERM) by employing the previously reported estrogen receptor (ER) partial agonist (**96**) as a lead candidate [57]. ER competitive binding assays and MCF-7 cell proliferation assays were used to evaluate the biological activities of the synthesized compounds. Among all tested compounds, compound **99** demonstrated the highest relative binding affinity when the position of the *N,N*-dimethylaminoethyloxy group was changed from para position to meta position. Moreover, compound **98** displayed the most potent ER-agonist activity (EC_50_: 1.4 nM) and the lowest maximal efficacy (E_max_: 50%) in MCF-7 cell proliferation assays. Overall, compound **98** (IC_50_: 0.4 M) inhibited 17β-estradiol-induced MCF-7 cell proliferation 10 times more effectively than lead candidate **96** (Scheme 24). Therefore, these compounds open a new window for scientists to design carborane-containing ER modulators or new therapeutic agents for ER-related diseases.

One of the most significant issues in modern clinical oncology is increasingthe therapeutic ratio. Boron neutron capture therapy (BNCT) is an unique therapeutic approach that aims to improve the therapeutic ratio for cancer cells, which have traditionally been difficult to treat. BNCT uses boronated chemicals to deliver boron-10 to tumors in a preferred manner, producing litihium-7 and an alpha particle after neutron irradiation. The alpha particle has a short range and selectively damages tumor tissues while protecting normal tissues [21].

**Scheme 25:**
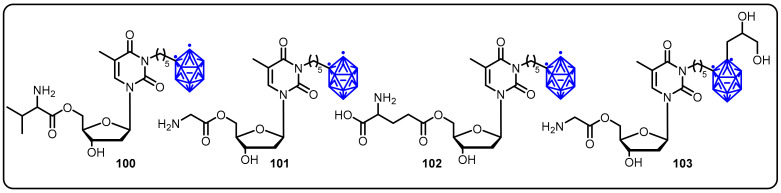
Prodrugs of 3-carboranyl thymidine analogues as antitumor agents.

Hasabelnaby et al. described improved strategies for the development of amino acid ester prodrugs of N5 and N5-2OH and evaluated them for boron neutron capture therapy (BNCT) of brain tumors [58]. The water solubilities of amino acid ester prodrugs were evaluated at different pH levels (5, 6, and 7.4) in phosphate-buffered saline (PBS), and the authors observed improved solubility—48–6600 times that of N5 and N5-2OH. Furthermore, the stability of prodrugs were tested in PBS at pH 7.4 with bovine serum and bovine cerebrospinal fluid (CSF). It was observed that 30-amino acid ester prodrugs were less sensitive to chemical and enzymatic hydrolysis than 50-amino acid ester prodrugs. Eventually, the N5 50-glutamate ester prodrug and N5 and N5-2OH 50-glycine ester prodrugs were found to be potential candidates for preclinical BNCT studies. (Scheme 25).

**Scheme 26:**
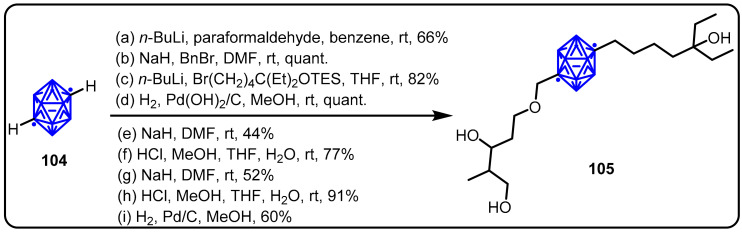
*p*-Carborane-based potent non-secosteroidal vitamin D analogues.

Non-secosteroidal vitamin D receptor (VDR) ligands are leading candidates in numerous biological applications. Fujii et al. demonstrated the synthesis and biological evaluation of carborane-based vitamin D analogues containing different substituents on the diol moiety. The synthesized compounds were tested for their VDR activity. Among the tested compounds, compound **105** exhibited the most potent vitamin D activity, with an EC_50_ value of 6.1 nM, comparable to the natural hormone 1α, 25(OH)_2_D_3_ (EC_50_ 4.4 nM) (Scheme 26) [59]. Compound **105** was one of the most potent non-secosteroidal VDR agonists and is a promising lead in the development of next-generation VDR ligands.

**Scheme 27:**
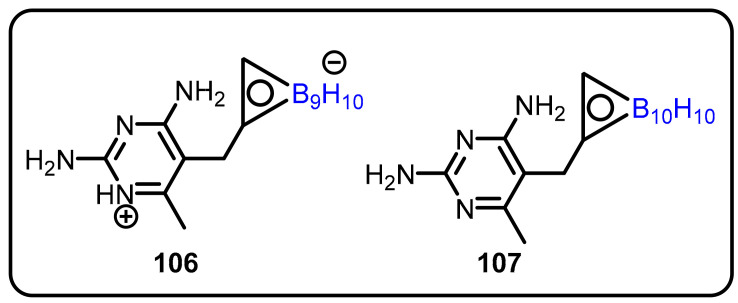
Boron-containing nonclassical antifolates.

The Borhani group designed and synthesized boron-containing, *ortho*-icosahedral carborane lipophilic antifolates and determined the crystal structures of their ternary complexes with dihydro-nicotinamide adenine dinucleotide phosphate and human dihydrofolate reductase (DHFR) [60]. The synthesized compounds were evaluated for inhibition of DHFR from different sources, including human or rat liver, *Mycobacterium* *avium*, *Pneumocystis carinii*, *Toxoplasma gondii*, and *Lactobacillus casei*, and showed IC_50_ values in the good to modest range ( 0.072–107 μM). The authors also tested these compounds for antibacterial activity against *M. tuberculosis* H37Ra, *L. casei*, and three *M. avium* strains, with MIC values in the range of 1.28 to 128 μM. In addition, cytotoxic activity was also evaluated against seven different tumor cells lines (Scheme 27). Compound **106** was further tested for BNCT, and tumor retention and selectivity ratios for boron distribution in tumor tissue versus normal tissue were low.

**Scheme 28:**
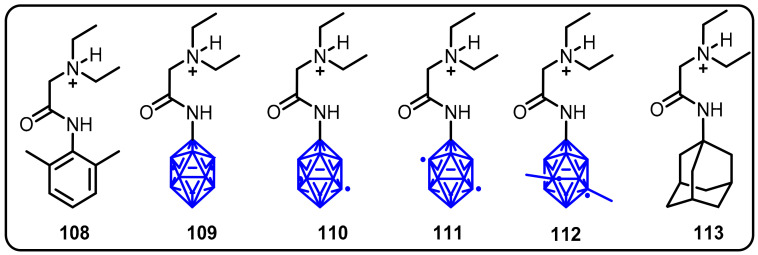
Carborane-derived local anesthetics.

Kracke et al. designed and synthesized novel anesthetic derivatives from lidocaine by replacing the phenyl ring of lidocaine with ortho-, meta-, C,C’-dimethyl meta-, and paracarborane clusters; the resulting compounds were identifiedas boronicaines [61].The boronicaine derivatives were tested for their analgesic activity and compared to lidocaine using a standardized method in mice following a plantar injection. The synthesized compounds demonstrated different analgesic activity in the following sequence: ortho-carborane (**109**) = C,C’-dimethyl meta-carborane (**111**) > para-carborane (**110**) > lidocaine (**108**) > meta-carborane (**112**) derivative (Scheme 28). Furthermore, ortho-boronicaine and C,C’-dimethyl meta-boronicaine provided analgesia for longer periods than lidocaine.

**Scheme 29:**
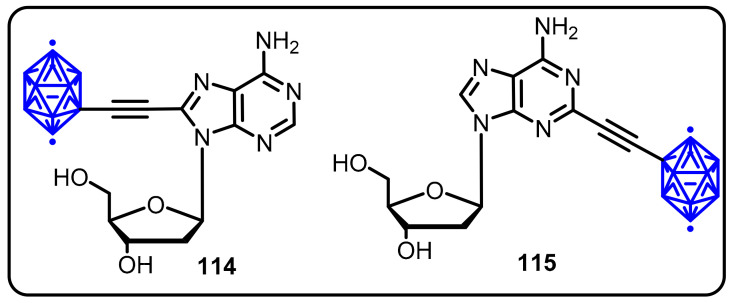
Antileukemic activity of novel adenosine derivatives bearing a boron cluster.

In 2016, Lesnikowski and colleagues revealed the synthesis of boron clusters containing adenosine derivatives. The synthesized compounds were tested for their cytotoxicity against primary peripheral mononuclear cells of 17 individuals with leukemias (16 CLL and 1 very rare PLL), and 5 healthy donors were used as a positive control. Two compounds (**114** and **115**) displayed potent in vitro cytotoxicity against chronic lymphocytic leukemia (CLL) and lymphocytic leukemia (PLL) cells at 15–20 µM, although normal cells from healthy donors were resistant (Scheme 29) [62]. Furthermore, when compared to control cells, compounds **114** and **115** displayed excellent activity in the proteolysis of the apoptotic indicators PARP-1 and lamin B1, DNA fragmentation, and induction of certain altered expression of the Mcl-1 protein apoptosis regulator.

**Scheme 30:**
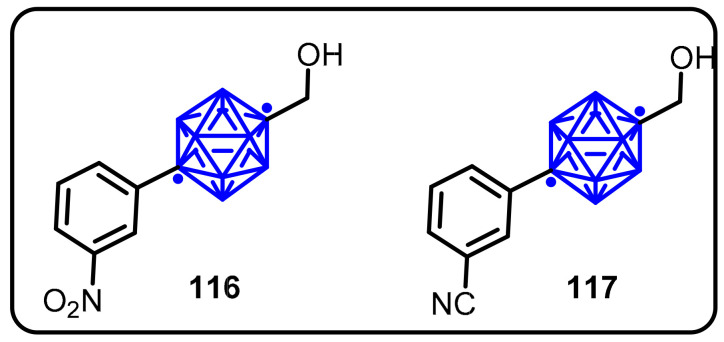
Androgen antagonists based on carborane as a hydrophobic core.

Endo and colleagues proposed the application ofcarboranes as a novel hydrophobic core structure in physiologically active compounds that hydrophobically interact with receptors [63]. The authors synthesized a series of carborane-based compounds and tested for antiandrogenic activity. Two compounds (**116** and **117**) demonstrated antiandrogenic activity greater than hydroxyflutamide in a reporter gene assay and a cell growth inhibition assay (Scheme 30). The unique character of carborane moiety likely provides distinctive characteristics of distribution and metabolism in vivo.

Hypoxia-inducible factor (HIF-1α) is a transcription factor that controls metastasis, glucose uptake, angiogenesis, invasion, and cell survival during cancer growth [102,103,104,105]. The HIF-1α and HIF-1β heterodimeric complex binds to specific nucleotide sequences to activate various hypoxia-responsive genes. Overexpression of HIF-1α is related to a poor prognosis and treatment resistance in cancer patients. Therefore, HIF-1α is considered to be a promising target for antitumor drugs.

**Scheme 31:**
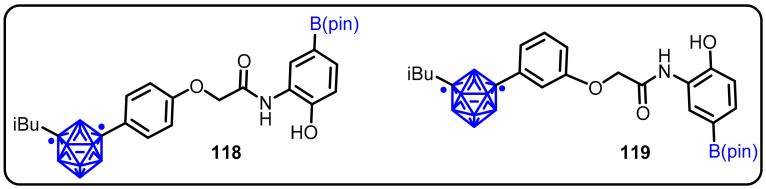
*m*-Carborane-containing phenoxyacetanilides as inhibitors of hypoxia-inducible factor.

Working on this hypothesis, Nakamura et al. developed various carborane analogues of LW6 (an adamantine derivative) as HIF-1α inhibitors [64]. HIF-1 transcriptional activity was examined using a cell-based dual-luciferase reporter gene assay in HeLa cells under hypoxia. Meta-carborane-containing phenoxyanilides (**118** and **119**) showed considerable inhibitory activity against hypoxia-induced HIF-1 transcription, with the IC_50_ values of 0.73 and 0.55 mM, respectively (Scheme 31).

### 2.4. BODIPY and Other Functionalities

**Scheme 32:**
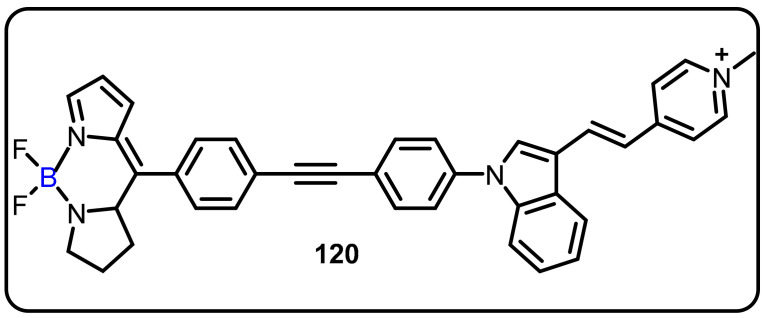
Bifunctional mitochondriatargeted as ananticancer agent.

In recent years, mitochondria have emerged as novel therapeutic targets for anticancer therapy due to the organelle’s important role in energy production, reactive oxygen species (ROS) generation, programmed cell death regulation, and calcium homeostasis. Therefore, effort has been applied toward the discovery of new multifunctional anticancer agents that could be utilized for simultaneous monitoring, treatment, and direct visualization of cancer. The Liu group reported a novel bifunctional mitochondria-targeted anticancer agent FPB (**120**). Compound **120** was evaluated for in vitro anticancer activity and organelle imaging capability (Scheme 32) [39,106,107]. This compound selectively accumulated in cancer cell mitochondria and induced cell death, with observed IC_50_ values 2–20 times below those of normal cells. Moreover, FPB also displayed excellent fluorescent properties. The authors suggested that FBP could be used as a bifunctional anticancer drug capable of selective anticancer activity, as well as efficient subcellular imaging.

**Scheme 33:**
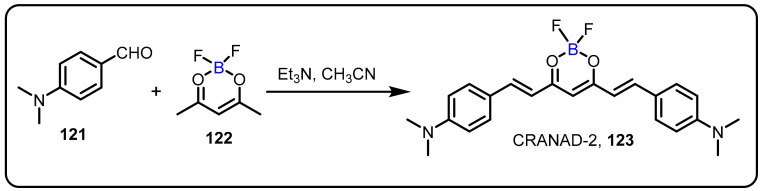
Difluoroboron-derivatized curcumins for in vivo detection of amyloid-β-deposits.

Alzheimer’s disease: 

Amyloid-β (Aβ) deposits have been recognized as a pathological hallmark of Alzheimer’s disease (AD). Ran et al. demonstrated the design and synthesis of CRANAD2 (**123**), a novelnear-infrared (NIR) Aβ plaque-specific fluorescent probe (Scheme 33) [65]. CRANAD-2 underwent a variety of modifications when it interacted with Aβ aggregates, including a 70-fold increase in fluorescence intensity, a 90nm blue shift (from 805 to 715 nm), and a high quantum yield. In addition, CRANAD-2 exhibited high affinity for Aβ aggregates (K_d_ = 38.0 nM), stability in serum, a reasonable log P value (log P = 3), and weak interaction with albumin. Furthermore, this probe was the first example of difluoroborate diketone compounds in vivo, offering a new NIR fluorescent dye fortissue, cell, and in vivo imaging in small animals. Interestingly, CRANAD2 exhibits all necessities for an NIR contrast agent for the detection of Aβ plaques in vitro and in vivo.

**Scheme 34:**
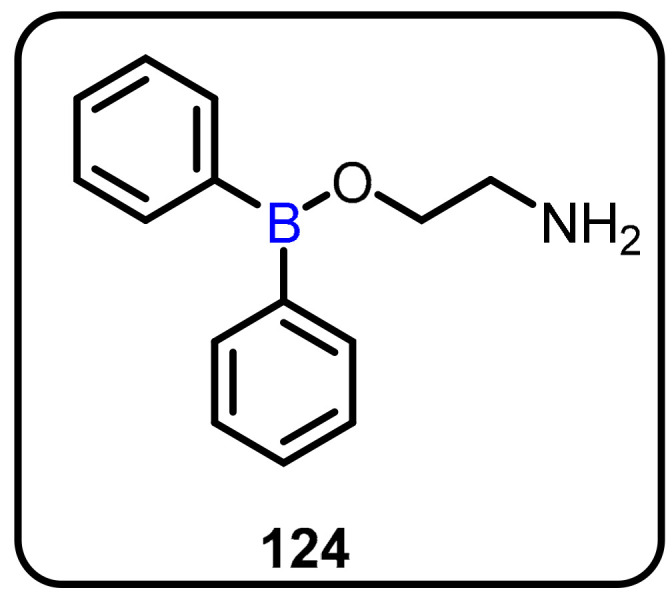
2-Aminoethoxydiphenyl borate for the treatment of allergic rhinitis.

Allergic rhinitis:

Allergic rhinitis (AR) is linked to a high rate of morbidity, mostly due to a decrease in quality of life and productivity worldwide. Antiallergic and antihistamine agents are commonly used in clinical treatment; however, they do not cure the illness. In this regard, Zheng et al. described an intervention in the Orai1 pathway to control IgE-mediated allergic reactions using AR mouse models [66]. The authors used Western blotting and real-time reverse transcription–polymerase chain reaction assays to determine Orai1 expression in the nasal mucosa and nasal-associated lymphoid tissue (NALT) of normal, control, and 2-aminoethoxydiphenyl borate (**124**) (2-APB)-treated mice. (Scheme 34). The findings revealed that Orai1 in the nasal mucosa and NALT enhanced in allergic conditions and decreased after the pathway was inhibited. 

Arginase inhibitor (erectile dysfunction):

L-arginine concentrations are maintained for NO synthase activity when arginase is inhibited. As a result, human penile arginase could be used as a target for the treatment of erectile dysfunction. Nitric oxide (NO) is a key regulator of erectile function, regulating nonadrenergic, noncholinergic (NANC) neurotransmission in the smooth muscle of the penile corpus cavernosum, resulting in fast relaxation and erection [108]. NO synthase, which catalyzes the oxidation of L-arginine to create L-citrulline and NO, is a crucial enzyme in smooth muscle functioning.

**Scheme 35:**
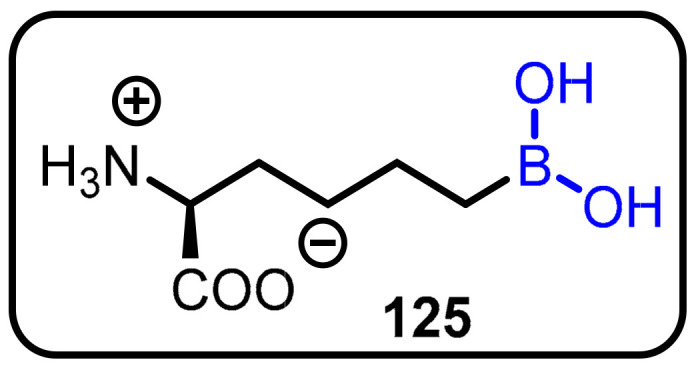
2(S)-Amino-6-boronohexanoic acid (ABH) used for the treatment of anthrax.

Boronic acids are effective inhibitors of hydrolytic enzymes, as they have an empty p orbital and, due to their electron-deficient nature, invite the addition of a protein-bound or solvent nucleophile to form a tetrahedral boronate anion that mimics the corresponding tetrahedral intermediate of a hydrolytic reaction [109,110,111,112]. The L-arginine analogue 2(S)-amino-6-boronohexanoic acid (**125**) (ABH; Kd = 0.11 mM; a stable boronic acid) has been the most potent arginase inhibitor identified to date (Scheme 35).

Anthrax: 

*Bacillus anthracis* is the causative agent of anthrax. *Bacillus anthracis* has established metabolic pathways that contain arginase, an enzyme that converts L-arginine to ornithine and urea. The nitric oxide (NO•) producedfrom the metabolism of L-arginineis principally responsible for *B. Anthracis* survival. Rosen et al. demonstrated 2(S)-Amino-6-boronohexanoic acid (ABH) as a potential inhibitor of bacterial arginase in endospore strains of *B. anthracis* for the first time [67] (Scheme 35).

**Scheme 36:**
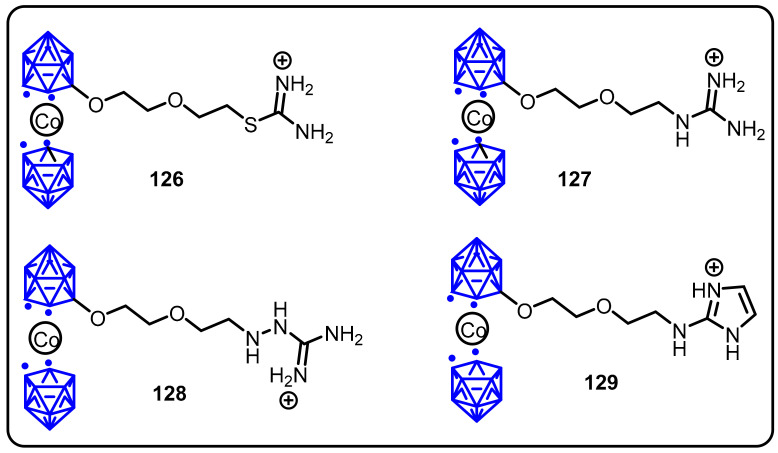
Nitric oxide synthase activation and inhibition by metallocarboraneclusters.

Nitric oxide (NO) is a biological messenger that is synthesized by nitric oxide synthases (NOSs), which are responsible for regulating various processes, includingimmune response, neurotransmission, and hemodynamic control. Therefore, in this regard, many inhibitors have been developed to precisely regulate the production of NO. Roman and colleagues introduced a novel strategy for the development of NOS isoform-specific inhibitors based on the use of perfectly substituted boron clusters. (**126**–**129**, Scheme 36) [113].

The antioxidant ability of boron compounds demonstrates its effect on lymphocyte proliferation. Oxidative stress prevents the cell cycle from progressing from the G0 to G1 phase, and it inhibits the proliferative response of human lymphocytes to phytohaemagglutinin. In actively immunized animals, the antigen, OVA, can stimulate the helper (CD4) and cytotoxic (CD8) T-cell subsets [114,115,116,117,118]. Ali et al.,using uninfected animals, observed no increase in CD8 cells in boron-treated mice. However, unlike CD8 cells, the number of CD4 cells increased significantly when mice were treated with borax. The increase in CD4 cells indicated an improved Th response and increased the function of the microphase, which is a major part of the immune system in preventing infection and has been used to identify the immune priming effect of borax [119].

**Scheme 37:**
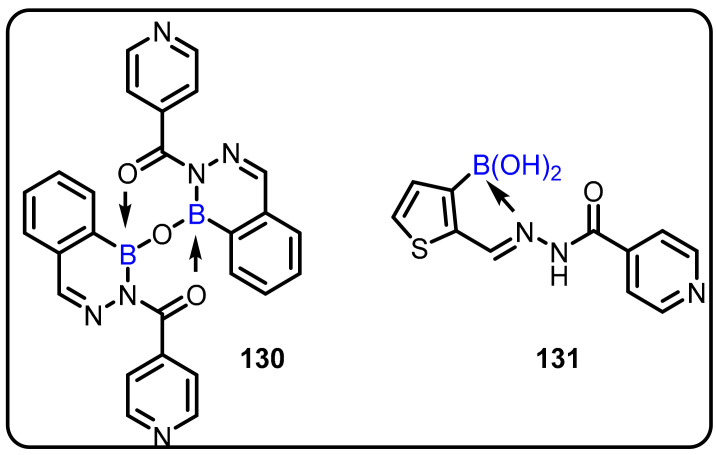
2-Acylated 2,3,1-benzodiazaborines as an anti-bacterial agent.

The Xu group described the synthesis of 2-acylated 2,3,1-benzodiazaborines and boron-based heterocycles. The authors demonstrated the reaction of acylphenylboronic acids with hydrazides, which led to the formation of novel 2-acyl-2,3,1-diazaborine heterocycles (**130** and **131**) with unusual hydration/dehydration reactivities. The newly synthesized compounds were evaluated for their antibacterial activity against *Mycobacterium smegmatis* and *Escherichia coli*. Among the tested compounds, two compounds (**130** and **131**) were found to be most potent against *M. smegmatis* and *E. coli*, with MIC values in the range of 2 to 32 µg/mL (Scheme 37) [54].

**Scheme 38:**
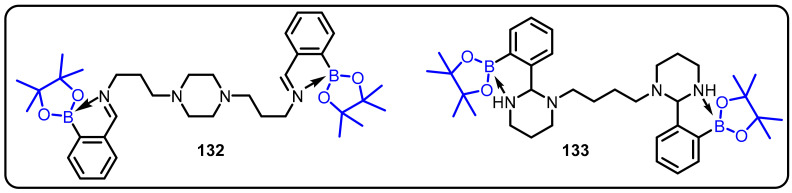
Amino boron compounds as an antit-uberculosis agents.

Westcott and colleagues discovered that relatively simple amino boron compounds could have a significant impact on the growth of *M. tuberculosis* [32]. The amino boron derivatives designed and synthesized in the process exhibited appreciably stronger anti-mycobacterial activity (**132** and **133**, Scheme 38). The enhanced activity observed for the more lipophilic derivatives can be explained by the increased ability of these probes to interact with their cellular target in *M. tuberculosis.*

**Scheme 39:**
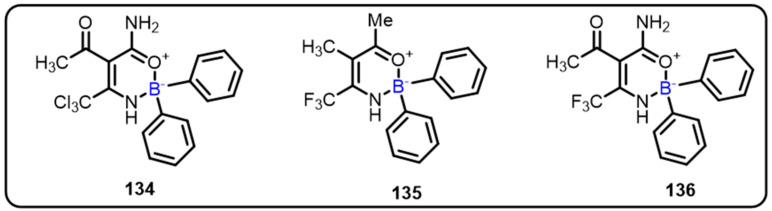
Oxazaborones as NLRP3 inflammasome inhibitors.

NLRP3 inflammasome inhibitors:

Oxazaborines are boron-based heterocycles, which have been proven to be an important motif in the creation of new NLRP3 inflammasome inhibitors. One of the major factors responsible for triggering chronic inflammatory responses is improper NLRP3 inflammasome stimulation, which ultimately leads to catastrophic diseases, such as type II diabetes, Alzheimer’s disease, gout, and atherosclerosis [120].Existing inhibitors, such as 2-aminoethoxy diphenylborinate (2APB), not only display potent inhibitory activity but also have effects on Ca^+2^ homeostasis. In this context, Baldwin et al. designed and synthesized oxazaborine compounds. Among all tested compounds, compound **134** displayed the best inhibition IC_50_ = 574 nM (IL-1β release inhibition) and in vivo efficacy without altering Ca^2+^ homeostasis (Scheme 39) [121]. In 2018, the same group demonstrated novel boron-based compounds and evaluated their potential for NLRP3 inhibitory activity.The insertion of steric and lipophilic substituents on the phenyl rings decreased the biological activity; however, the substitution of 4-F groups retained NLRP3 inhibitory effect, according to structure–activity relationships (SAR). Furthermore, SAR demonstrated that any phenyl ring substitution did not affect activity. However, when the lipophilic CCl_3_ group was replaced with the CF_3_ group on the oxazaborine rings (**135** and **136**), there was a loss of activity (Scheme 39). These promising findings could be vital for the development of novel oxazaborine-based heterocycles as effective anti-inflammatory drugs [68]. 

## 3. Conclusions

Boron-based inhibitors have a wide range of activity against a variety of amino acid residues with nucleophilic side chains. Medicinal chemistry has been fine tuning synergistic binding opportunities in the design of boron-containing molecules for cases where more than one residue is available in the binding site of a protein. The orientations between these motifs typically feature boron in an anionic tetrahedral form; however, depending on the protein host and the Lewis acidity of boron, a neutral trigonal planar configuration can also be realized. The Lewis acidity of boron can be controlled with new synthetic advancements in boron-containing heterocycles to inhibit binding to external nucleophiles. The rapid growth of the chemical genetics field will facilitate the exploitation of more boron chemicals in the future for target identification and validation steps in drug discovery. The old dogma that boron-based compounds are toxic and unstable has been overturned by substituting particular pharmacophore groups and chemical modifications as proof of concept. The FDA has already approved four boron-based drugs (Velcade, tavaborole, crisaborole, and ixazomib), and many others are in pipelines (Table 1). These successes have generated much interest for the boron chemistry community and many pharmaceutical companies. It will be important to determine which boron-based compounds can cross the blood–brain barrier (BBB), which could open a new avenue of research for neuroscience and neurodegenerative diseases, as has been amply described in recent review articles [122,123,124].

## Figures and Tables

**Table 1:** Summary of the disease indications associated with boron-based compound therapies.

	Boronic Acid/Esters/Aminoboronic Acids	Benzoxaborole Derivatives	Carboranes	BODIPYand Other Boron Reagents	Ref
Lipogenic inhibitors (heart)	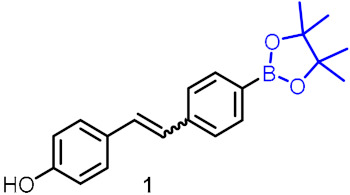				[22]
Improves lipid homeostasis	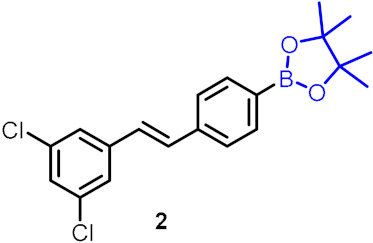				[23]
Amyotrophic lateral sclerosis	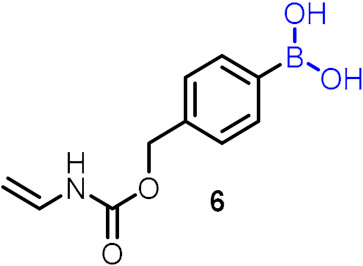				[24]
Proteasome inhibitor	Bortezomib (**7**) Ixazomib (**8**) Delanzomib (**9**)				[25,26,27]
Inhibitors of HIV reverse transcriptase (RT)	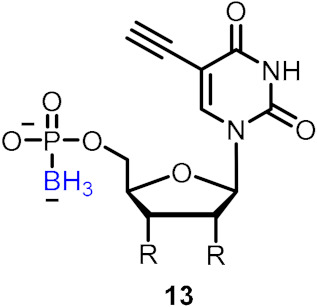				[28]
Inhibitors of human neutrophil elastase (HNE)	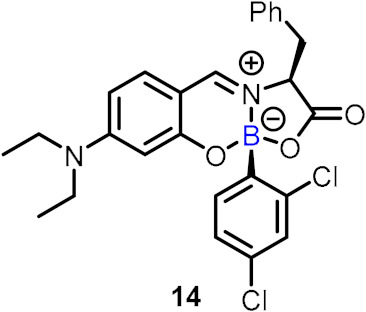				[29]
Anti-mycobacterial agents	Boromycin (**24**)		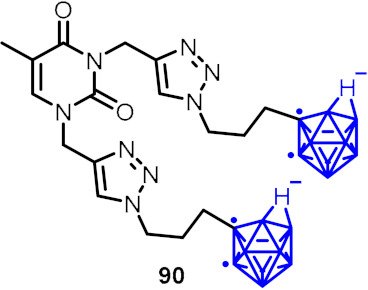	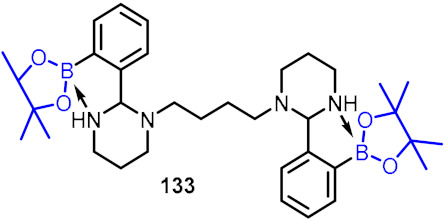	[30,31,32]
Inhibitor of HCV	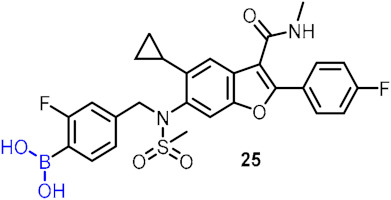				[33]
Anticancer agent	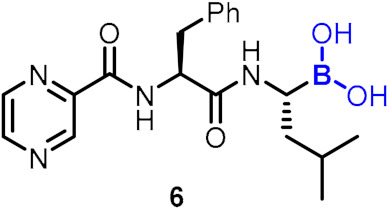			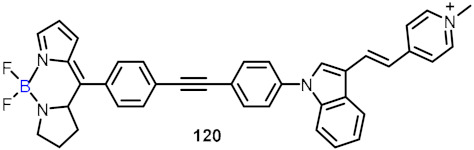	[34,35,36,37,38,39]
Protease inhibitor	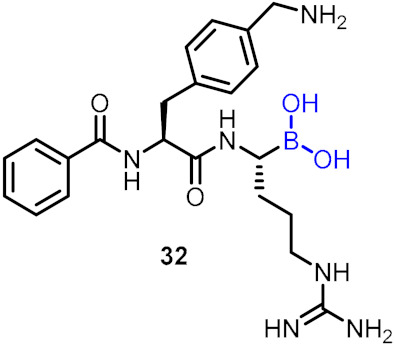				[40]
Antimalarial agents		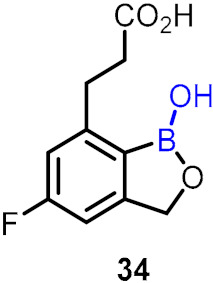			[41,42,43,44,45]
Animal ectoparasiticide		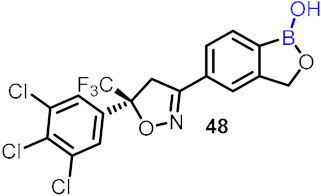			[46]
Anti-trypanosomal agents		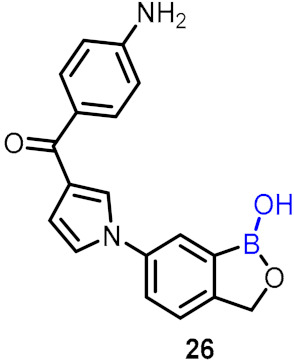			[47]
Anti-inflammatory diseases		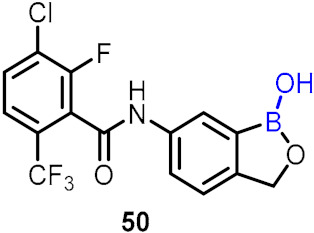	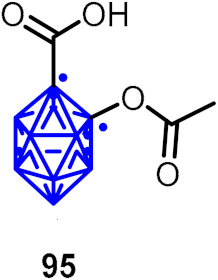		[48,49,50]
Antifungal drug		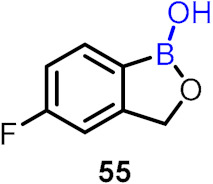			[51]
Anti-pneumococcal agent (lung)		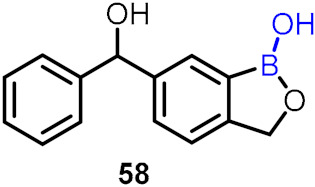			[52]
Antibacterial activity		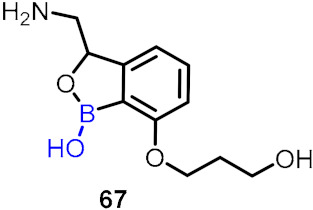		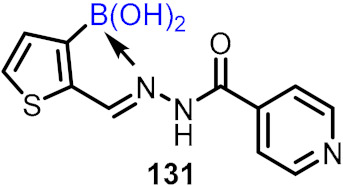	[53,54]
β-lactamase inhibitors		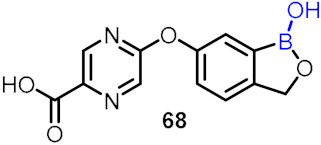			[55]
Antidepressant activity			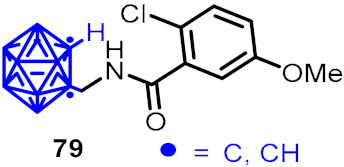		[56]
Estrogen receptor partial agonist candidates			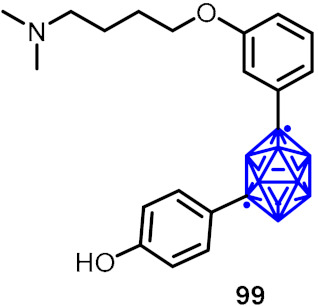		[57]
Brain antitumor agents			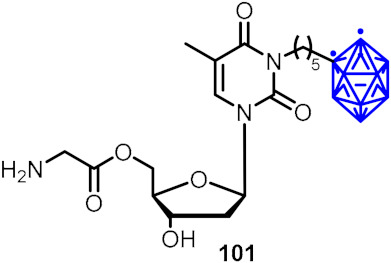		[58]
Non-secosteroidal vitamin D receptor			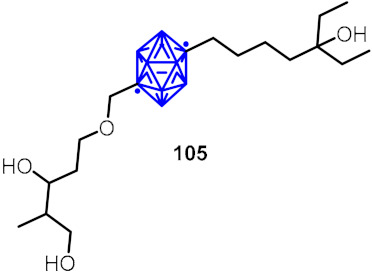		[59]
Antifolate analog			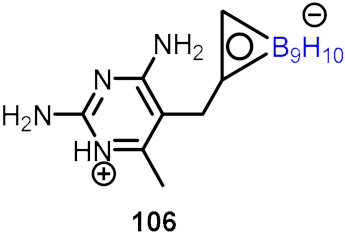		[60]
Anesthetics			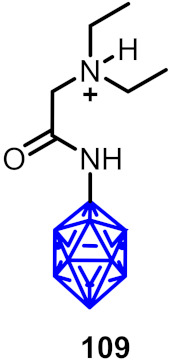		[61]
Antileukemic activity			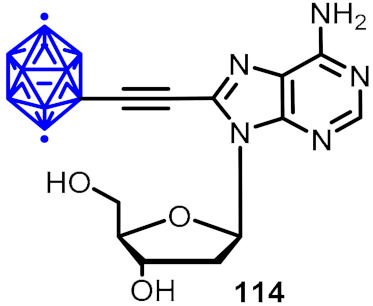		[62]
Androgen receptor antagonists			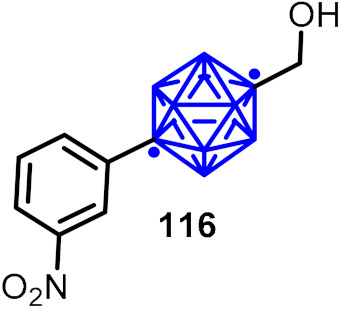		[63]
Hypoxia-inducible factor (HIF-1α) inhibitor			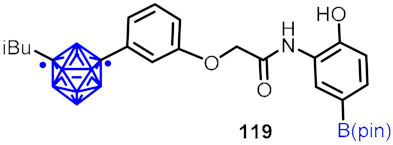		[64]
Amyloid-β detection				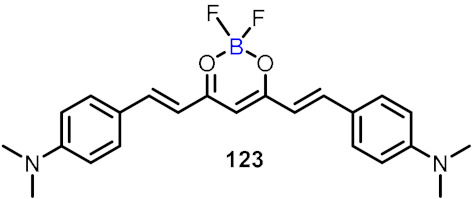	[65]
Antiallergic activity				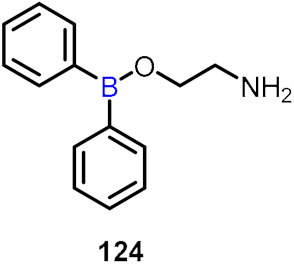	[66]
Anthrax				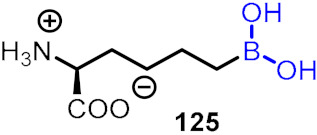	[67]
NLRP3 inhibitor				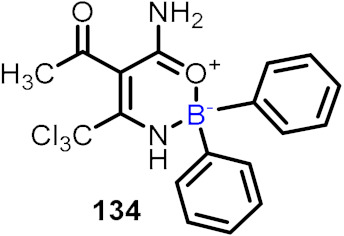	[68]

## Data Availability

Not applicable.
